# Novel Xanthone Derivatives Impair Growth and Invasiveness of Colon Cancer Cells In Vitro

**DOI:** 10.3390/biom11101480

**Published:** 2021-10-07

**Authors:** Jakub Rech, Daniel Sypniewski, Dorota Żelaszczyk, Natalia Szkaradek, Wojciech Rogóż, Anna Waszkielewicz, Henryk Marona, Ilona Bednarek

**Affiliations:** 1 Department of Biotechnology and Genetic Engineering, Faculty of Pharmaceutical Sciences in Sosnowiec, Medical University of Silesia, 40-055 Katowice, Poland; dsypniewski@sum.edu.pl (D.S.); ibednarek@sum.edu.pl (I.B.); 2 Department of Bioorganic Chemistry, Faculty of Pharmacy, Jagiellonian University Medical College, 30-688 Krakow, Poland; dorota.zelaszczyk@uj.edu.pl (D.Ż.); n.szkaradek@uj.edu.pl (N.S.); anna.waszkielewicz@uj.edu.pl (A.W.); henryk.marona@uj.edu.pl (H.M.); 3 Department of Physical Pharmacy, Faculty of Pharmaceutical Sciences in Sosnowiec, Medical University of Silesia, 40-055 Katowice, Poland; wrogoz@sum.edu.pl

**Keywords:** colorectal cancer, xanthones, angiogenesis, migration, invasion, metastasis

## Abstract

Natural xanthones are a large group of compounds from which promising anticancer properties could be further developed by chemical modifications. This study aimed to investigate the influence of four novel xanthone derivatives based on a naturally occurring xanthone skeleton on the invasiveness of colon cancer cells in vitro. First, the concentrations required to inhibit growth of three colorectal cancer cell lines to 50% (GI_50_) of all the studied compounds, as well as the natural xanthones used as a reference (gambogic acid and α-mangostin), have been established (MTS reduction test). Next, the assays determining several aspects of the GI_25_ xanthones influence on colorectal cancer cells, including cytotoxicity, migration and invasion potential, interaction with extracellular matrix and endothelial cells, as well as expression of selected invasiveness related genes have been performed. Our results demonstrate that these novel xanthone derivatives impair colorectal cancer proliferation, motility, adhesion to extracellular matrix and to endothelial cells, and also induce apoptosis and cell death. Moreover, their activity is comparable to cisplatin and 5-fluorouracil, used as reference compounds. Conducted research indicates our compounds for further research and development as novel drugs in colorectal cancer treatment.

## 1. Introduction

Colorectal cancer is the fourth most common tumor and a leading cause of cancer deaths worldwide. In 2018, around 1.4 million new diagnoses and nearly 810,000 related deaths were reported [1,2]. Moreover, there is an increasing tendency in the number of colorectal cancer patients, especially in societies with high or very high human development index, accounting for two-thirds of all colorectal cancer cases and 60% of deaths [1]. This is due to many unfavorable factors affecting the global population, including increasing age, genetic predispositions, as well as dietary and lifestyle factors, as the most significant [3,4].

Current therapies for colorectal cancer combine advanced chemotherapy, radiotherapy, and surgery, depending on tumor progression [5,6]. However, the insufficient treatment efficiency of invasive stages of colon cancer is a major cause of morbidity in patients suffering from this tumor type [3]. Tumor invasion is determined by such pivotal processes as cancer metastasis and angiogenesis. These processes are complex, multistage, and involve many subtle alterations in cellular phenotype, such as motility, adhesion and invasion through the extracellular matrix (ECM), interaction with endothelial cells, or epithelial–mesenchymal transition [7,8]. Among the angiogenic factors, vascular endothelial growth factor (VEGF) has been proven to be the key stimulator of tumor angiogenesis. One of the most important stimulators of VEGF expression is hypoxia-inducible factor 1α (HIF1α), which exerts a strong influence on the downstream of VEGF under the hypoxic conditions found in developing tumors [9].

Recently, a great amount of novel anticancer drug research has been conducted investigating naturally occurring plants used in folk medicine. Much attention has been given to xanthones, found mostly in higher plants, displaying numerous biological activities (e.g., antimicrobial, anticancer, cardioprotective, antioxidant, immunoregulatory) [10]. Two common xanthones are α-mangostin (MAG) and gambogic acid (GA) found in *Garcinia mangostana* fruit and *Garcinia hanburyi* resin, respectively [11,12]. GA is currently under clinical phase II study [13]. Both of these xanthones have been proven to significantly impair the growth and invasiveness of numerous cancer types, including breast, ovarian, cervical, lung, gastric, colon, prostate, melanoma, leukemia, and glioma [11,14,15,16,17]. To improve the anticancer activity of natural xanthones, many chemically synthesized xanthone derivatives have been obtained based on the promising results obtained from the screening of natural xanthones [18,19,20,21,22].

The aim of this study was to determine the influence of synthesized and naturally occurring xanthones (MAG, GA) on the growth and invasiveness of various colon cancer cell lines. After the treatments, cell cultures were submitted to assays estimating colon cancer cells’ tumorigenicity based on characteristics such as apoptosis, proliferation, clonogenicity, migration, invasion, adhesiveness, interaction with endothelial cells, and expression of selected invasiveness-related genes.

## 2. Materials and Methods

### 2.1. Chemicals

Acetic acid, acrylamide, bis-acrylamide, agarose, ANX (anisomycin), Brij-35, bromophenol blue, calcium chloride, cisplatin, Coomassie Brilliant Blue, crystal violet, DAPI (4′,6-diamidino-2-phenylindole dihydrochloride), DMSO (dimethyl sulfoxide), EDTA, 5-fluorouracil (5-FU), GA, gelatin, glycerol, glycine, MAG, methanol, paraformaldehyde, potassium hexacyano-ferrate (II) trihydrate, potassium hexacyano-ferrate (III), potassium phosphate monobasic (KH_2_PO_4_), potassium phosphate dibasic (K_2_HPO_4_), sodium dodecyl sulfate, sodium chloride, Tris, triton X-100, and zinc sulfate were purchased from Sigma-Aldrich. Bradford reagent was purchased from Fermentas. All media and chemicals used for in vitro cell cultivation (DMEM, FBS, Ham’s F-12, McCoy’s 5a, Dulbecco’s phosphate-buffered saline (D-PBS), trypsin-EDTA, gentamicin, hydrocortisone), CellTrace™ CFSE Cell Proliferation Kit, human recombinant epidermal growth factor (EGF), Gel-Red, and Geltrex LDEV-Free Reduced Growth Factor Basement Membrane Matrix Gibco™ were purchased from ThermoFisher Scientific (Waltham, MA, USA). Matrigel^®^ (BD Matrigel Cellware Basement Membrane) was purchased from Becton-Dickinson, Erembodegem, Belgium. Camptothecin and dexamethasone were purchased from Cell Signaling Technology.

### 2.2. Synthesis and Purification of Xanthone Derivatives

The detailed methodologies for the synthesis and purification of aminoalkanolic xanthone derivatives were described in our previous paper, along with their physicochemical properties. All compounds (Comp. **1**–**4**) were subjected to ^1^H and ^13^C nuclear magnetic resonance (NMR) and mass spectrometry analyses. The melting points of all compounds were also determined [23]. All compounds used in the study are summarized in Table 1.

### 2.3. Cell Culture and Treatment Conditions

All analyses were carried out on colorectal adenocarcinoma cell lines: Caco-2 (ATCC^®^ HTB-37™), HT-29 (ATCC^®^ HTB-38™), and LoVo (ATCC^®^ CCL229™). Adhesion and tube formation assays were carried out on the HMEC-1 cell line (ATCC^®^ CRL-3243™; a dermal microvascular endothelial cell line). Cell cultures were routinely propagated in a Hera-Cell humidified incubator (Heraeus, Kendro, Warsaw, Poland) at 37 °C/5% CO_2_. Cells were grown in appropriate media (DMEM for Caco-2, McCoy’s 5a for HT-29, and Ham’s F-12 for LoVo) supplemented with 10% FBS and 50 µg/mL gentamicin sulfate. Endothelial cells were grown in MCDB 131 medium additionally supplemented with EGF (10 ng/mL), hydrocortisone (1 μg/mL) and L-glutamine (10 mM). Based on solubility, xanthones were dissolved either in water or in DMSO, and stored at −20 °C. Directly before analysis, stock solutions were diluted to obtain the working concentrations. Treatments were performed for 12 h if not described otherwise. Additionally, several compounds were used as positive controls: recombinant human EGF (20 ng/mL for 2 h), ANX (4 μM for 5 h), and a mixture of apoptosis inducers containing 200 nM camptothecin, 200 nM etoposide, and 20 nM dexamethasone (for 6 h). Each experiment was carried out in triplicate. For MTS, apoptosis, and proliferation, 15,000 tumor cells were seeded into each well of the 96-well microplates. Other assays (adhesion, invasion, ELISA, molecular analyses, wound healing) were performed with initial cell density at 100,000 cells per well in 24-well microplates. Cells were grown overnight, and fresh media containing the studied xanthones were added the following day.

### 2.4. Cell Viability and GI_50_ Values Determination

Cell viability was assessed using a commercial test (CellTiter^®^ 96 Aqueous One Solution Cell Proliferation Assay, Promega, Madison, WI, USA) based on the reduction of a substrate dye in living cells to produce a brightly orange-colored formazan derivative (MTS assay). All analyses were carried out in 96-well plates according to the manufacturer’s instructions. After 2 h incubation at 37 °C, the intensity of the colorimetric reaction was measured by a Triad LT (Dynex, Chantilly, VA, USA) microplate reader at 450 nm. Compound concentrations required to reduce growth rates of cells to 50% (GI_50_) were determined based on MTS assay, performed in cell cultures after 24 h incubation with the range of serial dilutions of each tested compound.

### 2.5. Cell Proliferation

Cell proliferation was estimated based on 5-ethynyl-2′-deoxyuridine (EdU) incorporation using Click-iT™ EdU Alexa Fluor 488 Imaging Kit (ThermoFisher Scientific, Waltham, MA, USA), according to the manufacturer’s protocol. Cells were seeded in 96-well culture plates, transfected, and treated as indicated. To quantify the percentage of EdU-positive cells, poll images were captured by an inverted fluorescence microscope (Nikon Eclipse Ti, Warsaw, Poland). Cell nuclei were stained with DAPI. Ten vision fields were photographed and analyzed in each culture well (at least 100 cells in each well). Proliferation rate was established by quantification of EdU incorporation (Alexa Fluor 488-positive cells) vs. the total cell number (DAPI-positive cells) and expressed as a percentage of untreated cultures. All compounds were used at GI_25_ concentrations. ANX served as a positive control inhibitor of DNA synthesis.

### 2.6. Apoptosis Determination

The direct detection of apoptotic cells was performed by microscopic analysis. Cells were seeded in 96-well culture plates and treated as indicated. Cell cultures were stained with FITC-Annexin V and propidium iodide using a commercial kit (ThermoFisher Scientific, Waltham, MA, USA). All analyses were carried out under an inverted fluorescence microscope (Nikon Eclipse Ti). In each culture well, 10 vision fields were photographed and analyzed (at least 100 cells in each well). All compounds were used at GI_25_ concentrations. Additionally, a mixture of etoposide, camptothecin, and dexamethasone was used as a positive control to induce apoptosis according to different mechanisms of action.

### 2.7. Clonogenic Assay

The long-term effects of xanthone treatment on cancer cells colony formation were studied by clonogenic assay. Cells were seeded in six-well plates at 2000 cells per well, treated as indicated, and rinsed with fresh medium. After seven days, cells were fixed (4% paraformaldehyde), stained with 0.5% crystal violet, and photographed. All compounds were used at GI_25_ concentrations.

### 2.8. Wound Migration Assay

Cell motility was estimated by wound migration (healing) assay. Cells were seeded in 24-well plates to reach 90% confluency and treated as indicated. Then, in each well an injury line was made by applying a pipette tip across the center of the well to produce a 1 mm-wide wound area. Unattached cells were removed by washing with D-PBS. Cells were allowed to migrate for 24 h. The kinetics of migration within the wound lines were visualized under an inverted microscope (Axiovert 40CFL, Zeiss, Göttingen, Germany) at indicated time points. EGF served as a positive control.

### 2.9. Invasion Assay

Cell invasion was analyzed using 8 µm membrane inserts coated with Geltrex^®^ Basement Membrane Matrix in 24-well plates. Coating was performed directly before analysis using 100 μL diluted Geltrex (8 mg/mL) per insert, and coated inserts were incubated at 37 °C to allow gelling. Control (uncoated) inserts were included in each analysis to estimate the migration rate. An amount of 0.5 mL of xanthone-treated or control cell suspensions (5 × 10^4^ cells/mL in serum-free medium) were seeded on each membrane; lower chambers were filled with 0.75 mL of medium with normal serum supplementation. After 24 h of incubation, membranes were fixed in 4% paraformaldehyde, stained with 0.1% crystal violet, and analyzed under an inverted microscope. Non-invading cells were removed from the upper side of membranes with a cotton swab before staining. The invasion index was calculated as the number of invaded cells divided by the number of migrated cells. EGF served as a positive control.

### 2.10. Adhesion to ECM

Cell adhesion to ECM was determined in 96-well plates coated with Matrigel^®^ (BD Matrigel Cellware Basement Membrane, Becton-Dickinson, Erembodegem, Belgium). Cell suspensions from xanthone-treated and control cultures were added to each well (0.1 mL), and the plates were incubated for 2 h at 37 °C. Then, unattached cells were removed by washing with D-PBS, and adherent cells were fixed with 4% paraformaldehyde for 30 min, stained with 0.1% crystal violet, and counted under an inverted microscope (Axiovert 40CFL, Zeiss, Göttingen, Germany).

### 2.11. Adhesion to Endothelial Cells

The ability of tumor cells to adhere to endothelial cells was determined using HMEC-1 monolayers. HMEC-1 cells were seeded in 96-well plates and grown to reach 90% confluency. Xanthone-treated or control tumor cells were stained directly in culture plates with CellTrace™ CFSE (2.77 ng/mL, ThermoFisher Scientific (Waltham, MA, USA) for 15 min at 37 °C. Then, the CellTrace™ was removed and complete media were added. Cells were incubated for 30 min at 37 °C to allow the hydrolysis of the CellTrace™. Next, tumor cells were trypsinized, washed, centrifuged, and counted. Meanwhile, HMEC-1 cultures were stained with DAPI (5 min) and washed with D-PBS. Then, 3 × 10^4^ tumor cells in 50 μL of 2% growth media were added onto HMEC-1 cell monolayers and incubated for 1 h at 37 °C. Nonadherent cells were washed off with D-PBS and then cells were fixed with 4% paraformaldehyde. The number of adherent tumor cells was evaluated using an inverted fluorescence microscope (Nikon Eclipse Ti).

### 2.12. Capillary Tube Formation Assay

In vitro angiogenesis was evaluated using a capillary tube formation assay in Matrigel-coated 96-well plates (BD Matrigel^®^ Cellware Basement Membrane, Becton-Dickinson, Erembodegem, Belgium). For this, 3 × 10^4^ of HMEC-1 cells were seeded in each well and grown for 48 h. Cells were maintained in media containing an equal mixture of HMEC-1 medium and conditioned media prepared from the xanthone-treated cancer cell cultures. To obtain the conditioned media, 0.4 mL of cell culture supernatants were collected and centrifuged (3000× *g*, 5 min) to remove cell debris. Media collected from EGF-treated cell cultures served as positive controls. Tube formation was visualized under an inverted microscope (Nikon Eclipse Ti). To allow fluorescence observation, cells were stained with calcein-AM (0.5 μM, 15 min). For the semi-quantitative analysis, the angiogenic score was determined according to previous works [24].

### 2.13. RNA Extraction

Total RNA was extracted with TRI-Reagent^®^ (Sigma-Aldrich, Saint Louis, MO, USA) and phenol-chloroform according to the manufacturer’s protocol. All RNA extracts were digested with DNase I and purified of genomic DNA using a commercial kit (Direct-zol™ RNA MiniPrep, Zymo Research, Irvine, CA, USA). RNA concentration was spectrally determined by measuring absorbance at 260 nm (BioPhotometer, Eppendorf, Hamburg, Germany). RNA integrity was estimated by 2% agarose gel electrophoresis with GelRed^®^ staining.

### 2.14. Real-Time RT-PCR

Gene expression was determined by real-time RT-PCR with SYBR Green. mRNA levels were normalized by ∆∆Cq method with the use of untreated cultures as calibrators and HPRT mRNA as endogenous control. One-step real-time RT-PCR assay was carried out using a Mx3000P thermal cycler (Stratagene, San Diego, CA, USA). Reaction mixtures consisted of 12.5 μL of 2x Brilliant II SYBR Green RT-PCR Master Mix, 1 μL of reverse transcriptase, 0.3 μM of each sense and antisense primer, 0.1 μg of unknown RNA template, and water to a total volume of 25 μL. All reagents were purchased from Stratagene. The thermal profile was 50 °C for 30 min (reverse transcription), then 95 °C for 10 min, 40 two-step cycles of 94 °C for 15 s and 60 °C for 30 s, and 72 °C for 10 min (real-time PCR), followed by a dissociation protocol (60–95 °C, 30 min). The sequences of all primer sets used in the study are summarized in Table 2. Amplification products were separated in 2% agarose gels and visualized using GelRed^®^ staining. The specificity of PCR products was additionally confirmed by determination of their dissociation curves after each real-time RT-PCR amplification.

### 2.15. ELISA

Concentrations of soluble VEGF and intercellular adhesion molecule 1 (ICAM-1) were determined in cell culture supernatants by ELISA assays (Abcam, Cambridge, Great Britain). The assays were performed according to the manufacturer’s instructions. Absorbance at 450 nm was measured by a Triad LT (Dynex, Chantilly, VA, USA) microplate reader. For absolute quantitation, VEGF and ICAM-1 standards were used to obtain the standard curves in each assay.

### 2.16. Statistical Analysis

Quantitative data were compared by Student’s *t*-test or Mann–Whitney U test. For multiple comparisons, ANOVA or ANOVA Kruskal–Wallis were used; *p* < 0.05 was considered significant. All calculations were performed with Statistica v. 12 software (StatSoft Polska, Cracow, Poland).

## 3. Results

### 3.1. Cytotoxic, Antiproliferative, and Proapoptotic Potential of Xanthone Derivatives

The direct cytotoxic effects of the studied xanthones were established by GI_50_ (recognized as 50% growth inhibiton) estimation based on MTS assay. Colon cell cultures were treated with a range of linear dilutions (200–3.13 µM) of each compound for 24 h. Additionally, reference natural xanthones (MAG, GA) and common chemotherapeutic drugs (5-FU, cisplatin) were included in the analysis. All compounds exerted significant cytotoxic activity toward the studied colon cancer cell cultures, as reflected by their GI_50_ values (Table 3). Relative to the other cell lines, LoVo was the most resistant in the study, as indicated by the highest GI_50_ values for cisplatin and 5-FU. In contrast, xanthones exerted similar cytotoxicity to all the studied cell lines; however, HT-29 cells displayed relatively higher GI_50_ values of the synthetic xanthones, indicating that this cell line was the most resistant to the xanthone treatments. Previously our compounds were also subjected to cytotoxicity tests to non-tumor cell line HEK-293 and L929 indicating no cytotoxicity in GI_25_ (data not shown) [23].

**Figure 1:**
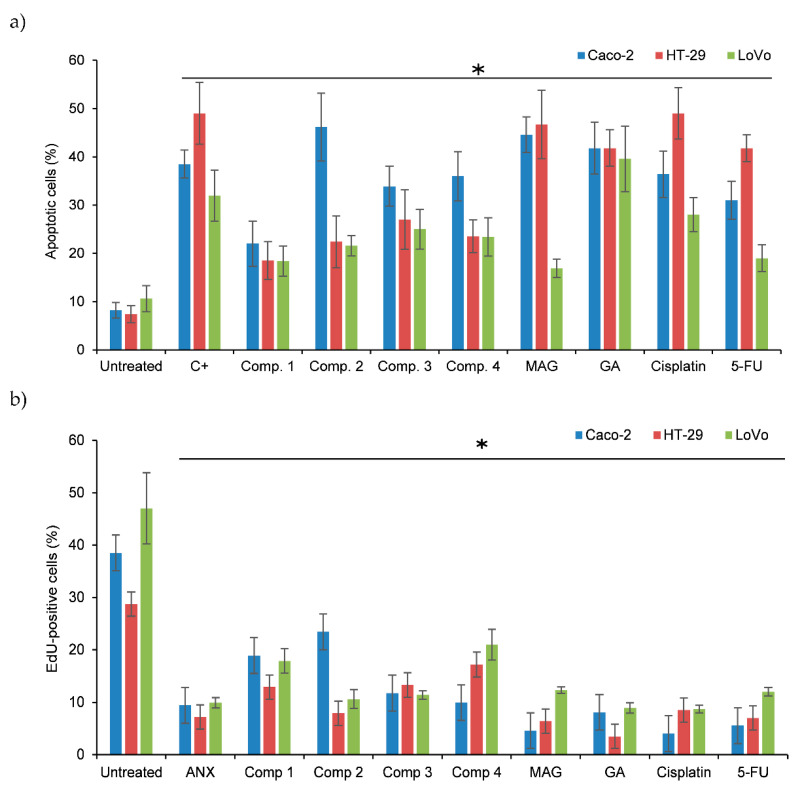
Antiproliferative and proapoptotic potential of xanthone derivatives. (**a**) Proliferation of xanthone-treated colon cancer cells. (**b**) Results of the microscopic evaluation of apoptosis and necrosis in the xanthone-treated cell cultures. Columns present the mean values (+/− S.D.) of dead cell numbers determined in three independent experiments, each in triplicate. C+: mixture of etoposide, camptothecin, and dexamethasone. * indicates a significant difference (*p* < 0.05) vs. untreated controls.

Based on the determined GI_50_ values, noncytotoxic concentrations were established for the subsequent assays. For proliferation and apoptosis assays, all treatments were performed for 12 h at the GI_25_ concentrations of the studied compounds. Proliferation was evaluated microscopically by the incorporation of the nucleotide analogue EdU into the DNA of actively proliferating cells. Nuclei were counterstained with DAPI for the calculation of total cell number in each vision field. Analysis of microscopic images revealed that all xanthones significantly decreased the proliferation of the tumor cell lines compared to untreated controls, and their antiproliferative potential was comparable with that of ANX, which served in this study as a control inhibitor of cell proliferation (Figure 1a). Similar results were obtained for each tumor line.

The same treatment conditions were used to evaluate the influence of these compounds on apoptosis in the studied cell lines. Microscopic analysis confirmed that all xanthones stimulated a statistically significant induction of apoptosis-type cell death in Caco-2 and HT-29 cell lines, while LoVo cells were more resistant to apoptosis induction (Figure 1b). The strongest effects in all cell lines were exerted by GA. The reference chemotherapeutic drugs (cisplatin and 5-FU) displayed similar proapoptotic and antiproliferative potential compared to the studied xanthones, suggesting that the xanthone treatments could be effective in colon cancer therapy.

### 3.2. Xanthone Treatments Impaired the Clonogenicity, Migration, and Invasion of Colon Cancer Cells

**Figure 2:**
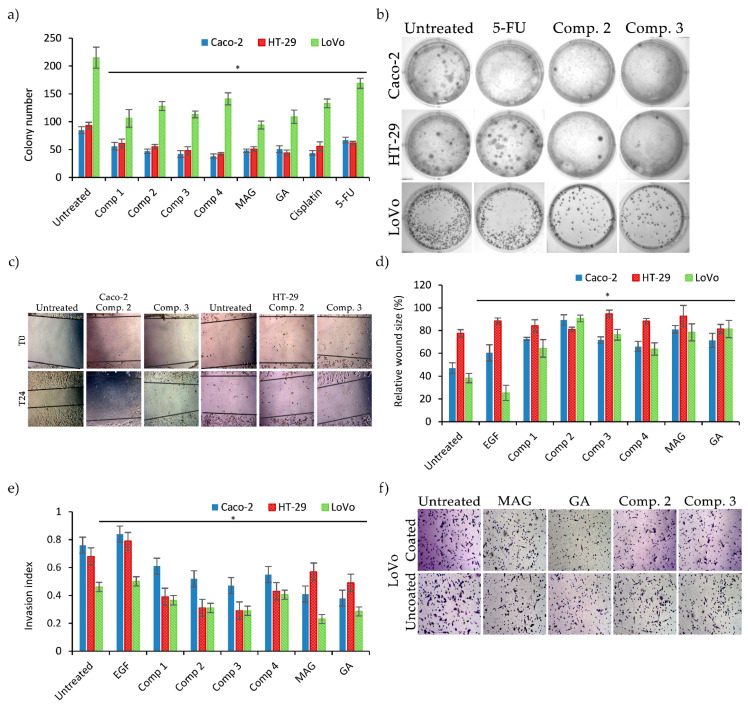
Antimetastatic activity of xanthones. (**a**) Results of clonogenic assays presented as the numbers of single colonies in culture plates. (**b**) Representative images of clonogenic assay (**c**). Representative images of the wound healing assay. (**d**) Migration of colon cancer cells estimated by the wound healing assay. (**e**) Invasion index values calculated for colon cancer cells after xanthone treatments. (**f**) Representative images of invasion assay on LoVo cell line. Graphs present mean values (+/− S.D.); * indicates a significant difference (*p* < 0.05) vs. untreated controls.

Next, we analyzed the potential of xanthone derivatives to impair the clonogenicity of colon cancer cells. There was a clear difference between the clonogenic potential of LoVo and those of other cell lines, as seen in Figure 2a,b. Comp. **1**–**4** presented similar proliferation-inhibiting properties, comparable to reference compounds. The most active xanthone in HT-29 and Caco-2 cells was Comp. **4**, but it also had a minor influence on LoVo cells. On the contrary, Comp. **1** had the most potent antiproliferative effects in LoVo cells but had the least effect on Caco-2 and HT-29 cells. Moreover xanthone-treated cell colonies appeared to be smaller and fewer in number. In fact, GA and MAG demonstrated the highest potential among different colorectal cancer cell lines, and thus can be assessed as the most universal drugs evaluated in this study.

Subsequently, we investigated the influence of Comp. **1**–**4** on cell motility by wound healing assay. There was clear response (i.e., motility decrease) in both LoVo and Caco-2 cells treated by all compounds (Figure 2c,d). Comp. **2** presented the highest activity against these cell lines. On other hand, the motility of the HT-29 cells was significantly decreased only under treatment with Comp. **3**. This compound had a lesser influence on LoVo and Caco-2 cells. We also observed that HT-29 generally had a lower motility than other cell lines, and it was further reduced by xanthone derivatives treatment.

To further assess the potential of these xanthone derivatives, we evaluated their invasion index based on invasion trough Geltrex-coated inserts. EGF was used as a positive control to stimulate cell migration. Every tested compound decreased cell motility, and thus wound repair and invasion (Figure 2e,f). This was especially evident in Caco-2 and HT-29 cells. Comp. **1**–**4** were most active against HT-29 cells, strongly decreasing their invasion index. MAG and GA had a stronger influence on Caco-2 and LoVo cells, with a lesser effect on HT-29 cells.

### 3.3. Interaction between Colon Cancer, Endothelial Cells, and ECM under Xanthone Treatments

**Figure 3:**
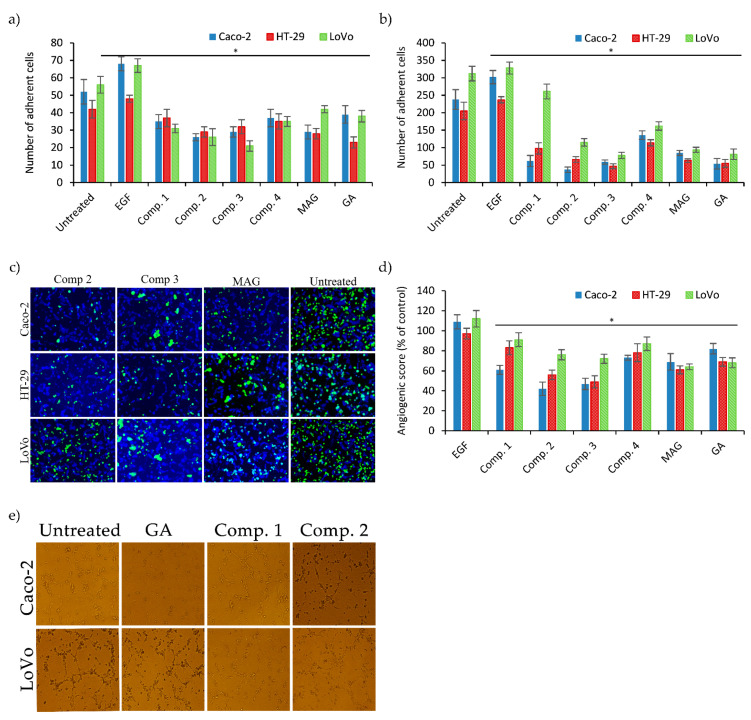
Cell–environment response after xanthone treatment. (**a**) Results of Matrigel adhesion assay. EGF served as positive control. (**b**) Quantification of tumor cell adhesion to endothelium. (**c**) Representative images of tumor cells (green fluorescence) adhered to HMEC-1 (blue fluorescence) monolayers (mag. 200×). (**d**) Results of capillary tube formation assay. Graph presentation of angiogenic score of Caco-2, HT-29, and LoVo cell lines after xanthone treatments. Results are expressed as the change in angiogenic score with respect to untreated controls. EGF was used as positive control (+). (**e**) Representative images of tube assay (mag. 200×). Graphs present mean (+/− S.D.) values; * indicates a significant difference (*p* < 0.05) vs. untreated controls.

The interaction between colon cancer cells and ECM was analyzed by adhesion assays using Matrigel-coated plates. The results are summarized in Figure 3a. Xanthone treatment led to a significant reduction in the adhesiveness of Caco-2 and LoVo cells compared to untreated controls. In both cell lines the strongest inhibitory effect was exerted by the synthetic xanthones, especially Comp. **2** and **3**. For HT-29 cells the influence of the xanthone treatment was the weakest, and only GA, MAG, and Comp. **2** significantly reduced cell adhesion to ECM.

To study the interaction between tumor and endothelial cells we performed adhesion assays as described in Section 2.11 and presented in Figure 3b,c. The number of colon cancer cells adherent to HMEC-1 monolayers was significantly higher for untreated and EGF-treated cells than for cells treated with xanthones. This effect was most noticeable for the Caco-2 cell line, where the highest decrease was observed. For xanthone-treated LoVo and HT-29 cells the strongest inhibition of cell adhesion was exerted by Comp. **3**, GA, and MAG; for Caco-2 Comp. **2** had the strongest influence. These results indicate that all the xanthones used in the study significantly impair the ability of colon cancer cells to interact with endothelial HMEC-1 cells.

Next, we analyzed whether the xanthone treatments influenced the capillary tubular phenotype of endothelial cells. HMEC-1 cells were cultivated on Matrigel-coated 96-well plates to allow cell tube formation. Then, supernatants collected from the xanthone-treated colon cancer cultures were added to the HMEC-1 cultures (1:1 ratio). Tube formation was visualized using an inverted microscope and results were expressed as the angiogenic score values calculated according to the previous papers [24]. The addition of supernatants from Caco-2 cell cultures led to a significant decrease in tube formation by HMEC-1 cells after the xanthone treatments (Figure 3d). Similar results were observed in cultures exposed to the supernatants from HT-29 and LoVo cell cultures, with the exception of cultures treated with Comp. **1** and Comp. **4** (LoVo cultures), which did not lead to significant changes in tube formation compared to untreated controls. Overall, these results indicate that the studied xanthones significantly reduce the ability of endothelial cells to form capillary structures.

### 3.4. Xanthone Treatments Influence the Expression of Genes Involved in Invasion and Metastasis

**Figure 4:**
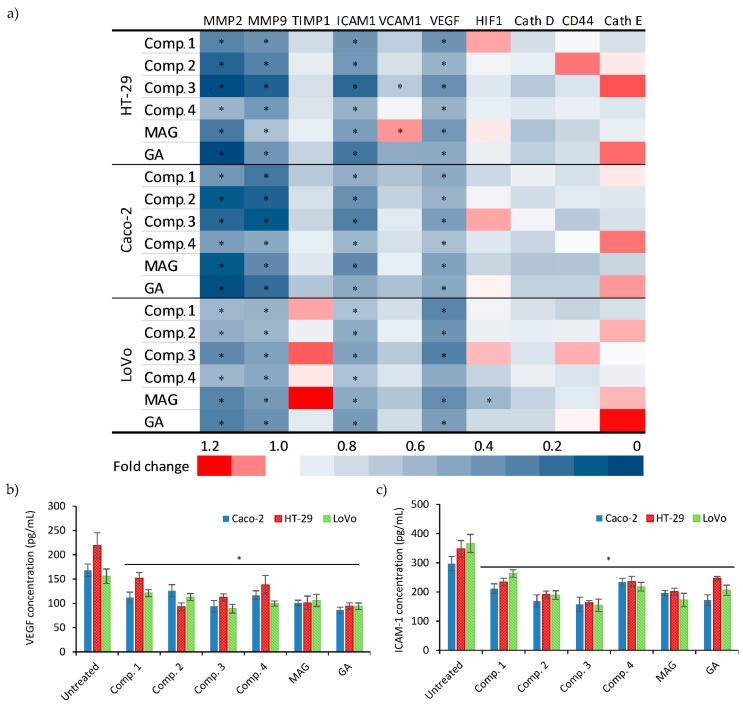
(**a**) Effect of xanthone treatment on the change in expression of selected genes evaluated by real-time RT-PCR assays performed in Caco-2, HT-29, and LoVo cell cultures after xanthone treatments. Results of ELISA indicating changes in (**b**) VEGF and (**c**) ICAM-1 concentration after treatment with corresponding compounds. Graphs present median (+/− S.D.) values; * indicates a significant difference (*p* < 0.05) vs. untreated controls.

The panel of the studied genes involved in migration/invasion and metastasis processes included matrix metalloproteinases 2 and 9 (MMP2, MMP9), tissue inhibitor of metalloproteinases 1 (TIMP1), cathepsin D (CTSD), VEGF, HIF1α, ICAM-1, vascular cell adhesion protein 1 (VCAM-1), homing cell adhesion molecule (HCAM, CD44), and E-cadherin (CDH1). The results are summarized in Figure 4a. MMP2, MMP9, and ICAM-1 mRNA expression were heavily decreased as a result of treatment with reference xanthones and xanthone derivatives. TIMP1 expression was also reduced but to a lesser degree, excluding the LoVo cell line, where TIMP1 was increased by a maximum factor of 1,2. These results correlate with those of the invasion assay and thus the decrease in cell motility and invasion. Additionally, VEGF was observed downstream, while HIF1α maintained its base expression. VCAM1 decreased significantly only in HT-29 cells treated with Comp. **3** and MAG. Other genes showed a slight decrease in expression, but this observation did not reach statistical significance.

### 3.5. Expression of VEGF and ICAM-1 Protein under Xanthone Treatment

Levels of VEGF and ICAM-1 released to cell culture supernatants were determined by ELISA. Results are presented in Figure 4b,c. ELISA confirmed that ICAM-1 expression significantly decreased in all xanthone-treated cell cultures in comparison to untreated controls, and the strongest inhibitory influence was displayed by Comp. **3**. Additionally, xanthone treatments led to a significant decrease in VEGF levels in all three cell lines. The highest inhibitory effect was observed in cultures treated with GA, and the most effective synthetic xanthones were Comp. **2** and **3**.

## 4. Discussion

Mangosteen xanthones, especially MAG and GA, have been proven to display significant chemo-preventive and anticancer properties against numerous tumor types [12,15,16,17,25,26,27]. Moreover, chemical modification of the xanthone structure is believed to further increase the anticancer properties, as described in [28,29]. Previously we reported the vast anticancer activity of these xanthone derivatives against breast, cervical, kidney, lung, and bladder cancer cell lines. We have also discussed the structure-activity relationship [23].

Due to an insufficient number of papers investigating the influence of xanthones on colon cancer, in this study we focused on the further evaluation of these properties in Caco-2, HT-29, and LoVo colon cancer cell lines. These cell lines were chosen based on their differences in origin (Caco-2 and HT-29—tissue of origin; LoVo—metastatic site) and characteristics (Caco-2—enterocyte-like cells; HT-29—goblet cells; LoVo—adenocarcinoma).

Natural xanthones isolated from *Garcinia mangostana*, especially MAG, induce cell cycle arrest and apoptosis, and decrease the proliferation of DLD-1 [30], COLO-25 [31], HT-29 [30,32], and HCT-15 [33] colon cancer cell lines. Chitchumroonchokchai et al. confirmed that MAG inhibits the proliferation of HT-29 cells and reduces the expression of BCL-2 and β-catenin [32]. Similarly, the cytotoxic effect of natural mangosteen xanthones and GA in colorectal cancer cells (LoVo) was confirmed by Fang and Yu [34,35]. Aisha et al. observed that MAG not only increased apoptosis but also impaired migration, insert invasion, and xenograft tumor growth in HCT-116 colon cancer cells [36].

In a similar synthesis-screening study, Lemos et al. showed that chemical modification of the xanthone ring yielded highly active xanthone derivatives. The obtained compounds disrupted the MDM2-p53 complex, suppressing the HCT-116 cell proliferation and decreasing the invasiveness and inducing cell-cycle arrest [28]. Isoalvaxanthone, a natural xanthone derivative isolated from plants, also had similar activities in SW620 colon cancer cells, additionally inhibiting Rac1 factor and subsequently reducing the expression and activity of MMP-2 and MMP-9 [37]. We also observed this activity in our novel xanthone derivatives when applied to Caco-2, HT-29, and LoVo cell lines. These compounds possess both short- and long-term cytotoxic activity, inducing apoptosis and necrosis and retarding the proliferation and clonogenic potential of cancer cells. In response to xanthones, cancer cells form fewer and smaller colonies in vitro. This may impair metastasis and tumor development. Similarly, the ability of MAG to impair tumor growth in vitro and to reduce xenograft metastases in mice has been confirmed in prostate [38] and breast [39] cancers. In our study, we confirmed that both proliferation and metastasis decreased in cancer cell lines treated with xanthones [12,30]. Metastasis is a common factor known to be responsible for 90% of cancer deaths. Moreover, distant metastasis of colon cancer significantly decreases the survival rate, even in high-income countries [3]. Xanthone derivatives led to a decrease in cell motility in every colon cancer cell line, in a similar manner to MAG and GA. This activity of MAG, γ-mangostin, and xanthone extract is also confirmed by other authors [23,36,40].

The tumor microenvironment has a profound impact on cancer, influencing cell proliferation, tumor progression, and invasive potential. Cancer invasiveness is a complex and multistage process composed of numerous changes in the cellular phenotype (e.g., cell motility, adhesion, and the expression of certain enzymes and signaling molecules). Enhancement of the cellular response, as well as modulation of the secretion of growth factors and certain cytokines, may contribute to the restriction of tumor development and metastasis, and to the improvement of treatment efficacy [41]. Metastasis is often dependent on the cell–ECM relationship, and further ECM cell infiltration. It was previously described that MAG decreased invasion through Matrigel-coated inserts and the adhesion of cancer cells to collagen-coated plates [42]. Here, we describe that the chemical modification of xanthone may lead to a decrease in the invasion index, as shown in HT-29 cells treated with Comp **2** and **3** (Figure 2e). To elaborate, our evaluation of colon cancer cell adhesion to ECM demonstrated the high inhibitory activity of our compounds in this regard. Similar to our work, others have also described similar results in the treatment of colon cancer cells with MAG and GA [37,43].

In parallel with the analysis of the adhesion of colon carcinoma cells to the ECM, we also analyzed their ability to adhere to microvascular endothelial cells (HMEC). Cancer cells may interact with HMEC monolayers in different ways: they can adhere to, create holes in, or penetrate through them [44]. Overall, the interactions between cancer cells and endothelial cells are crucial in the control of tumor invasiveness and the ability to create metastases. Our results indicate that the studied xanthone derivatives reduced the adhesion of colorectal cancer cells to the HMEC monolayers. Comp. **3** was particularly effective in the Caco-2 line.

In response to appropriate stimuli (e.g., bFGF, VEGF, PDGF, EGF), endothelial cells (primary or immortalized) form geometrical structures similar to capillaries. Capillary tube formation assay makes it possible to study how quickly angiogenesis proceeds in vitro. With this test we showed that xanthone derivatives were able to inhibit colorectal cancer cells’ ability to stimulate the formation of new blood vessels. Similarly, Shiozaki et al. confirmed the antiangiogenic activity of MAG using HUVEC migration and tubule formation assays [45].

ECM rearrangement by specific metalloproteinases reflects on tumor activity. In almost every cancer, tissue MMPs are upregulated, leading to easier and increased metastasis formation. The rapid degradation of native ECM leads to faster tumor growth and angiogenic formation. Further, it leads to a self-running closed circle, intensifying tumor growth endlessly [41]. Chen et al. confirmed that MAG reduces the level of MMP-9 mRNA through the suppression of the MEK/ERK signaling pathway and thus reduces renal cancer metastasis [46]. Likewise, Zhou and Ma described a decrease of invasion and migration as well as decreased MMP-2 and MMP-9 mRNA in SW620 colon cancer cells treated with GA [47]. We previously described a decrease in MMP-9 and MMP-2 mRNA expression and enzymatic activity in breast (MCF-7), cervical (HeLa), lung (A549), and bladder (T24) cancer cells treated by aminoalkanol xanthone derivatives [23]. This study also suggests that xanthone treatment led to the downregulation of MMP-2 and MMP-9 mRNA levels. Our studies clearly showed that xanthones had the ability to modify these parameters, particularly in Caco-2 and LoVo cells—very positive results were especially noted for Comp. **2** and **3**.

The VEGF pathway has a strong impact on colon cancer cell growth and proliferation [48]. A has been reported that MAG exerts antiangiogenic activity by inhibiting of phosphorylation of VEGF receptors and ERK [45,49]. Additionally, GA and its derivatives have been proven to suppress angiogenesis and tumor growth through the inhibition of VEGF receptor signaling [29,50]. In this study, we noted a very similar response of colon cancer cells treated with Comp. **1**–**4**, including the decrease in VEGF mRNA and receptor concentration together with maintained HIF1α mRNA expression.

Intercellular and vascular adhesion molecules (ICAM-1 and VCAM-1) play an important role in cancer progression; however, data in the literature are often contradictory. There are reports suggesting that ICAM-1 molecules enable cancer cells to avoid the immune system, enhance tumor growth, and angiogenesis [51,52]. On the other hand, it has been proven that ICAM-1 suppresses colon and ovarian cancer metastasis and growth [53,54,55]. VCAM-1 is a glycoprotein associated with endothelial cell adhesion. Its elevated expression may contribute to the acceleration of metastasis formation as well as angiogenesis and thus to increasing cell invasiveness [56]. Kawczyk-Krupka et al. indicate that anticancer treatment did not exert any effect on the ICAM-1- or VCAM-1-dependent cell adhesion of colorectal cancer cells [57]. In our study, a significant decrease in ICAM-1 expression under the xanthone treatment was observed, while no major changes were detected in VCAM-1 expression.

Further consideration is required regarding the utilization of drug delivery systems such as synthetic polymers and naturally occurring human proteins, which delay and extend the release of compounds yielding improved anticancer activity [58,59,60].

## 5. Conclusions

Our study evaluated the anticancer activity of novel synthetic xanthone derivatives. We presented the substantial cytotoxic properties of synthetic xanthone derivatives in comparison to naturally occurring xanthones (MAG, GA) and rutinously utilized cytostatics (5-FU, cisplatin). Moreover, our study proved that synthetic modifications of xanthones may increase the anticancer properties of currently known substances. Comp. **2** and **3** were the substances with the most promising anticancer activities, although there were clear differences in the response of different cell lines to these compounds. In our study, we present various evidences on the decrease in colorectal cell motility and invasiveness, and thus metastasis development risk. These novel xanthone derivatives are promising anticancer agents which require further attention and research.

## Figures and Tables

**Table 1:** Chemical structure of synthetic derivatives and natural xanthones evaluated in this study.

**Synthetic Xanthone Derivatives Structures**			
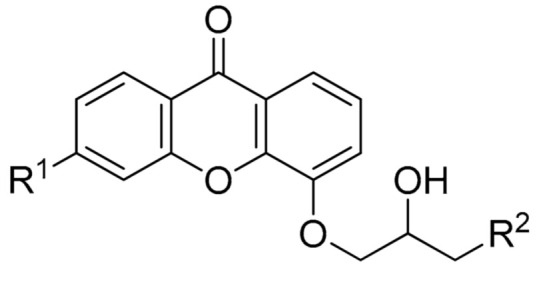			
Compound	R1	R2	Salt
Comp. **1**	-H	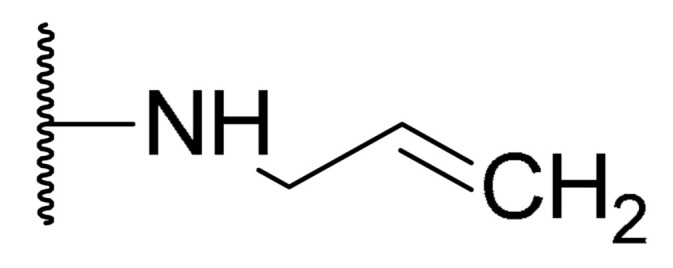	HCl
Comp. **2**	-Cl	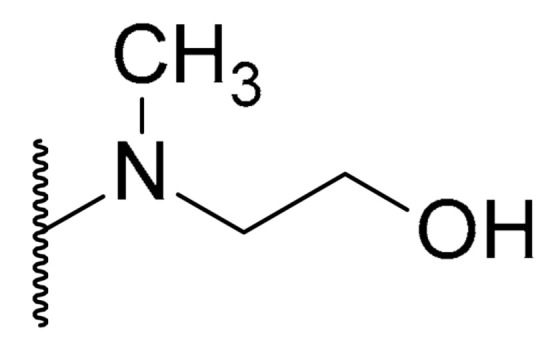	HCl
Comp. **3**	-Cl	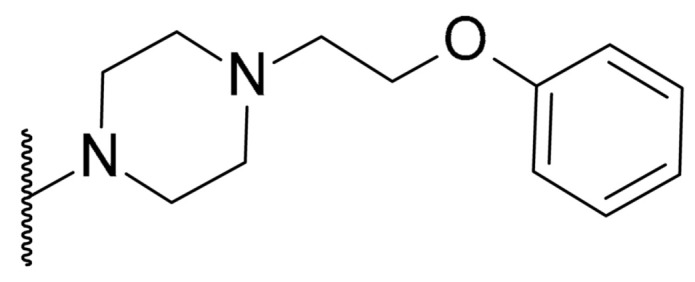	2HCl
Comp. **4**	-Cl	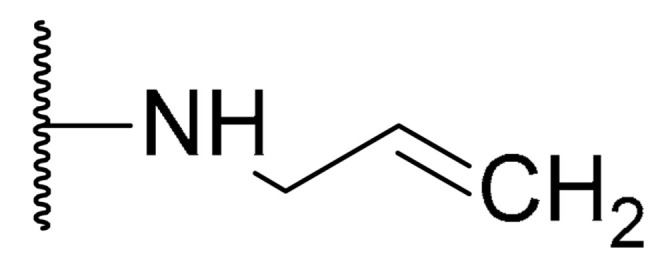	HCl
**Natural Xanthones Structures**			
MAG	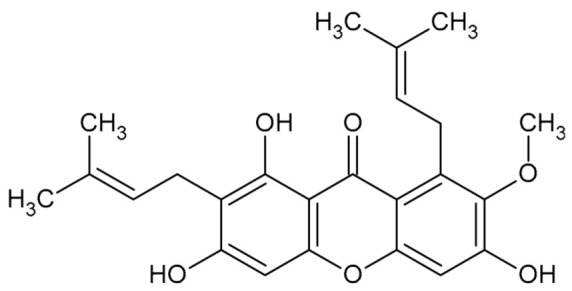		
GA	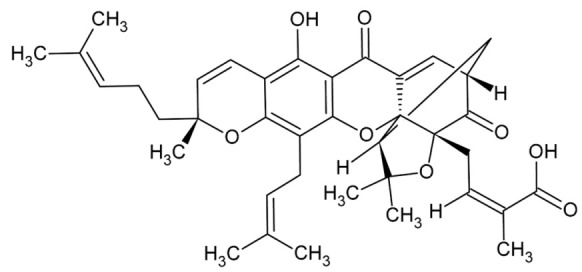		

**Table 2:** The sequences of primers used in the real-time RT-PCR analyses.

Target mRNA and Access Number	Sequences (Sense and Antisense)	Amplification Product Length
*CTSD* (NM_001909.4)	5′TTGCTGTTTTGTTCTGTGGTTTTC3′	60 bp
	5′CAGACAGGCAGGCAGCATT3′	
*CD44* (NM_000610.3)	5′GAAGATTTGGACAGGACAGGAC3′	225 bp
	5′CGTGTGTGGGTAATGAGAGGTA3′	
*CDH1* (NM_004360.4)	5′ATGGCTGAAGGTGACAGAGC3′	204 bp
	5′GAGGTTCCTGGAAGAGCACC3′	
*HIF1α* (NM_001243084.1)	5′CAAGAACCTACTGCTAATGCCA3′	188 bp
	5′TTTGGTGAGGCTGTCCGA3′	
*MMP-2* (NM_001302508.1)	5′CGCTCAGATCCGTGGTGAG3′	130 bp
	5′CATCAATCTTTTCCGGGAGCT3′	
*MMP-9* (NM_004994.2)	5′GCTCACCTTCACTCGCGTG3′	60 bp
	5′CGCGACACCAAACTGGATG3′	
*TIMP1* (NM_003254.2)	5′CTTCCACAGGTCCCACAACC3′	303 bp
	5′CAGCCCTGGCTCCCGAGGC3′	
*VEGF-A* (NM_001171623.1)	5′CTTGCCTTGCTGCTCTACC3′	200 bp
	5′CACACAGGATGGCTTGAAG3′	
*ICAM-1* (NM_000201.2)	5′GGCTGGAGCTGTTTGAGAAC3′	201 bp
	5′ACTGTGGGGTTCAACCTCTG3′	
*VCAM-1* (NM_001078.3)	5′AAGATGGTCGTGATCCTTGG3′	137 bp
	5′GGTGCTGCAAGTCAATGAGA3′	
*HPRT* (NM_000194.2)	5′CCTGGCGTCGTGATTAGTGA3′	135 bp
	5′CGAGCAAGACGTTCAGTCCT3′	

**Table 3:** Comparison of GI_50_ and GI_25_ values for the synthetic xanthone derivatives MAG and GA (reference xanthones), as well as the reference chemotherapeutic drugs cisplatin and 5-FU, as determined by MTS assay for colon tumor cell lines after 24 h treatment.

Compound	GI_50_ [μM]			GI_25_ [μM]		
	Caco-2	HT-29	LoVo	Caco-2	HT-29	LoVo
Comp. **1**	32.5	49.5	36.0	10.8	16.5	12.1
Comp. **2**	26.2	29.5	24.0	8.7	9.8	7.9
Comp. **3**	15.5	38.0	19.5	5.2	12.7	6.5
Comp. **4**	12.4	56.5	41.1	4.2	18.8	13.7
MAG	7.5	19.9	17.9	2.5	6.6	6.0
GA	13.6	25.4	12.5	4.5	8.5	4.2
5-FU	10.5	15.5	31.0	3.5	5.1	10.3
Cisplatin	14.0	19.0	41.5	4.6	6.3	13.8

## Data Availability

The data presented in this study are available on request from the corresponding author.
